# Selective prefrontal cortex responses to joint attention in early infancy

**DOI:** 10.1098/rsbl.2009.1069

**Published:** 2010-01-27

**Authors:** Tobias Grossmann, Mark H. Johnson

**Affiliations:** Centre for Brain and Cognitive Development, Birkbeck, University of London, London, UK

**Keywords:** social cognition, prefrontal cortex, infancy, joint attention

## Abstract

The process by which two people share attention towards the same object or event is called joint attention. Joint attention and the underlying triadic representations between self, other person and object are thought to be unique to humans, supporting teaching, cooperation and language learning. Despite the progress that has been made in understanding the behavioural importance of joint attention during early social development, almost nothing is known about the brain substrate that supports joint attention in the developing infant. We examined responses in five-month-old infants' prefrontal cortex during triadic social interactions using near-infrared spectroscopy. The results demonstrate that, even by the age of five months, infants are sensitive to triadic interactions and, like adults, they recruit a specific brain region localized in left dorsal prefrontal cortex when engaged in joint attention with another person. This suggests that the human infant is neurobiologically prepared for sharing attention with other humans, which may provide the basis for a wide variety of uniquely human social and cultural learning processes.

## 1. Introduction

Attending and responding to eye gaze is crucial for human social interactions. Specifically, eye gaze plays an important role in directing and coordinating attention during triadic interactions between self, other and the environment. During a typical triadic interaction, a person may establish eye contact with another person and then direct that person's gaze to an object or event. The psychological process by which two people share attention towards the same object or event is called joint attention. Joint attention and the underlying triadic representations (self–other person–object) are thought to be unique to our species, supporting teaching, cooperation and language learning (Tomasello *et al*. 2005; Saxe 2006; Csibra & Gergely 2009). Impairments in joint attention are one of the earliest signs of autism (Charman 2003).

More than 30 years ago, Scaife & Bruner (1975) first observed that infants follow a person's gaze and engage in joint attention. This seminal finding and the subsequent surge of behavioural research on the topic led to a rejection of the long-held Piagetian notion of infant egocentrism (Mundy & Newell 2007). Moreover, research on joint attention suggests that infants' ability to share a common point of reference develops before spoken language (Baldwin 1995), and that joint attention processes are important precursors of the later development of higher-level mental state attribution (Charman *et al*. 2001).

Despite the progress that has been made in understanding the behavioural emergence of joint attention during infancy (e.g. Carpenter *et al*. 1998), almost nothing is known about the brain substrate that supports joint attention in the developing child. Investigating the neural basis of joint attention in infants is important for several reasons, including the possibility that behavioural tasks are insufficiently sensitive to reveal early abilities in infants. In adults, joint attention relies on the recruitment of the medial prefrontal cortex (Williams *et al*. 2005; Schilbach *et al*. in press), a brain structure that has been more generally implicated in social cognition and theory of mind (Amodio & Frith 2006). Here, we examined haemodynamic responses in five-month-old infants' prefrontal cortex during triadic social interactions using near-infrared spectroscopy (NIRS) (see Lloyd-Fox *et al*. (2010) for a description of the method and its use with infants).

Infants were presented with three different experimental conditions. In all conditions, the infant watched an adult's face in the middle of the screen with an object either to the left or to the right side of the face. In the joint attention condition, the adult raised her eyebrows and smiled while holding eye contact with the infant, then shifted her eyes towards the object, then shifted her eyes back to the infant and finally turned her head towards the object. The ability to jointly attend with another person requires the infant to not only attend to the external object or event but also to monitor (i) the other person's attention towards the same object or event and (ii) the other person's attention in relation to the self. Our two control conditions thus disrupted these two critical aspects of joint attention. In the first control condition, the no referent condition, the person behaved exactly the same as in the joint attention condition, except that she looked and turned towards the side where there was no object. In the second control condition, the no eye contact condition, the person looked at the object without establishing any eye contact with the infant (the person looked down with her eyes closed before shifting her eyes towards the object). We predicted that the brain region that is specialized in dealing with triadic social interactions should be selectively engaged during the joint attention condition but not during the two control conditions, because they lack the triangular nature required to establish joint attention.

## 2. Material and methods

### (a) Participants

The final sample consisted of 15 five-month-old infants (eight girls) aged between 136 and 159 days (*M* = 149.1 days). An additional eight five-month-olds were tested but not included in the final sample because they had too many motion artefacts, resulting in too few usable trials for analysis (minimum number of four trials per condition). Please note that an attrition rate at this level is within the normal range for an infant NIRS study (Lloyd-Fox *et al*. 2010). All infants were born full-term (37–42 weeks gestation) and with normal birthweight (>2500 g). All parents gave informed consent before the study.

### (b) Stimuli and procedure

Animated photo-realistic face stimuli were generated using Poser 6.0 software (Curious Lab Inc., Santa Cruz, CA). Infants sat on their parent's lap while watching the stimuli on a computer monitor within an acoustically shielded, dimly lit room. The faces presented subtended a visual angle of 38° × 25°, and each eye subtended 3° × 5°). The experimental sessions consisted of 9 s long trials. The three experimental conditions were randomly distributed over the session with no more than two trials of the same condition occurring in a row. The inter-trial interval randomly varied between 8 and 12 s. Non-social moving visual stimuli were presented during the inter-trial interval to keep infants' attention.

### (c) Data acquisition and analysis

**Figure 1.**
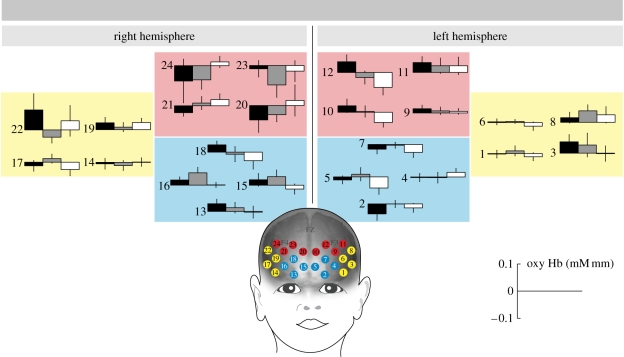
Prefrontal brain responses measured in five-month-old infants. This graph depicts mean oxygenated haemoglobin concentration changes (±s.e.m.) during the joint attention, no referent and no eye contact conditions measured from 24 NIRS channels within dorsal, ventral and lateral ROIs. Black shaded box, joint attention; light grey shaded box, no referent; white box, no eye contact; red, dorsal; yellow, lateral; blue, ventral.

Cortical activations were measured using a Hitachi ETG-4000 NIRS system. The multi-channel system uses two wavelengths at 695 and 830 nm. Two custom-built arrays consisting of nine optodes (five sources, four detectors) in a 12-channel (source–detector pairs) arrangement with an inter-optode separation of 25 mm were placed over the frontal lobe on each hemisphere using an Easycap (Falk Minow). The NIRS method relies on the optical determination of changes in haemoglobin concentrations in cerebral cortex which result from increased regional cerebral blood flow. NIRS data were continuously sampled at 10 Hz. For analysis, after calculation of the oxyHb concentration changes, pulse-related signal changes and overall trends were eliminated by low-pass filtering (Butterworth, fifth-order, lower cut-off 0.5 Hz). Movement artefacts were corrected by an established procedure (Koch *et al*. 2006; Wartenburger *et al*. 2007), which allows marking of artefacts and then padding the contaminated data segments by linear interpolation. Cortical responses were assessed by comparing average concentration changes (oxyHb) within trials (15 s after stimulus onset) between the experimental conditions. For statistical analyses, three regions of interest (ROIs: dorsal, ventral and lateral prefrontal cortex) were selected on the basis of prefrontal cortex anatomy and a meta-analysis of the adult functional magnetic resonance imaging (fMRI) work (Amodio & Frith 2006; see figure 1 for ROIs). An omnibus repeated measures analysis of variance (ANOVA) was conducted with within-subjects factors: ROI (3) × hemisphere (2) × condition (3). Subsequently, planned comparisons were performed using repeated measures ANOVAs for each ROI individually with within-subjects factors: channel location (4) × hemisphere (2) × condition (3).

## 3. Results

The omnibus ANOVA revealed a significant three-way interaction between ROI, hemisphere and condition, *F*_4,56_ = 3.201, *p* = 0.019, partial *η*^2^ = 0.186. Planned comparisons revealed a significant interaction between condition and hemisphere (*F*_2,28_ = 6.944, *p* = 0.004, partial *η*^2^ = 0.332) for the dorsal prefrontal region. The left dorsal prefrontal region was specifically sensitive to joint attention interactions. This region showed the predicted pattern of an increased brain response when the joint attention condition was compared with the no referent condition (*t*_14_ = 1.795, *p* = 0.047 one-tailed), and when the joint attention condition was compared with the no eye contact condition (*t*_14_ = 2.125, *p* = 0.026 one-tailed), whereas in the right dorsal prefrontal region, there was no difference between the joint attention and no referent conditions (*t*_1,14_ = 0.002, *p* = 0.494 one-tailed) and the response was significantly decreased in the joint attention when compared with the no eye contact condition (*t*_1,14_ = −1.969, *p* = 0.034 one-tailed). As shown in figure 1, within the left dorsal prefontal region, comprising channels 9, 10, 11 and 12, all channels showed increased responses to joint attention when compared with the other conditions. For channel 12 alone, this effect attained statistical significance (*F*_2,28_ = 3.962, *p* = 0.031, partial *η*^2^ = 0.281) (see table S1 in the electronic supplementary material for statistical results of all channels). No main effects or interactions were obtained in the planned comparisons performed for the ventral and lateral prefrontal regions.

## 4. Discussion

These results show that, even by the age of five months, infants are sensitive to triadic interactions and, like adults, they recruit a specific prefrontal region localized in left dorsal prefrontal cortex when engaged in joint attention with another person (Schilbach *et al*. in press). It is interesting to note that the infant brain response was lateralized to the left hemisphere, which is not only in line with the adult fMRI work (Schilbach *et al*. in press) but also concurs with findings showing that higher rates of resting glucose metabolism in left prefrontal regions measured with positron emission tomography, and synchronized brain activity measured from left frontal electrodes during resting EEG, are positively correlated with children's tendency to initiate joint attention bids in the second year of life (Caplan *et al*. 1993; Mundy Card & Fox 2000). The finding is also interesting in light of work showing that adults with autism have atypical brain structure (increased grey matter) in the same left prefrontal regions that are functionally implicated in joint attention in healthy adults (Waiter *et al*. 2004; Williams *et al*. 2005).

A critical distinction has been drawn between *responding to joint attention* (RJA) and the actual *initiation of joint attention* (IJA; Mundy & Newell 2007). In the current study, however, by focusing on how infants respond to joint attention initiated by an adult, we were not able to examine this potentially important difference. Schilbach *et al*. (in press) assessed the neurobiological basis of the distinction between RJA and IJA in adults, and found that while there are regions commonly involved in both kinds of joint attention, such as the left medial dorsal prefontal cortex, the ventral striatum appears to be specifically engaged only when an adult *initiates* joint attention. This specific involvement of the ventral striatum has been argued to be the basis of the positive affective experience associated with directing someone else's gaze. While future neuroimaging work with infants should address this important distinction between RJA and IJA, it is unknown whether NIRS can detect responses from brain structures located as deep as the ventral striatum (Grossmann 2008).

In conclusion, using a novel paradigm and a modern optical imaging technique well suited to studying freely behaving infants, we were able to demonstrate that parts of the left dorsal prefrontal cortex are selectively activated already very early in the development of joint attention. This brings into question claims that joint attention is an ability that only emerges towards the end of the first year of life (also termed the ‘nine-month-revolution’; Tomasello 1999). The current findings suggest that the human infant is neurobiologically prepared for sharing attention with other humans, and this may provide the basis of a wide variety of uniquely human social and cultural learning processes.
